# Refractory Dermatophytosis in a Spitz Dog Successfully Managed with Posaconazole: A Case Report

**DOI:** 10.3390/ani16071050

**Published:** 2026-03-30

**Authors:** Anisha Tiwari, Bhanu Kirti Khajuria, Curtis Plowgian, Cheol-Yong Hwang

**Affiliations:** 1 Suvidha Pet Care and Derma Centre, 1A/1A Trikuta Nagar, Jammu 180020, Jammu & Kashmir, India; 2 Suvidha Vet Care and Research Centre, 100/3 Sanjay Nagar, Jammu 180010, Jammu & Kashmir, India; suvidhavets@gmail.com; 3 Animal Dermatology Clinic, Indianapolis, IN 46250, USA; cplowgian@adcmg.com; 4 Department of Veterinary Dermatology, Seoul National University, Seoul 08826, Republic of Korea; cyhwang@snu.ac.kr

**Keywords:** antifungal therapy, dermatophytosis, posaconazole, spitz dog, refractory infection

## Abstract

Dermatophytosis is a superficial fungal skin disease of cats and dogs. The most common pathogens of small animals belong to the genera *Microsporum* and *Trichophyton*. It is an important skin disease because it is contagious and can be transmitted to people. Refractory dermatophytosis has become a disease of increasing concern in dermatological practice due to poor responses to standard antifungal therapy. The condition is characterised by chronicity, recurrence, or persistence despite adequate treatment. This report describes the clinical presentation and therapeutic management of refractory dermatophytosis in an 8-year-old intact male Spitz dog weighing 10 kg presenting with persistent alopecia, scaling, erythema, and pruritus despite multiple courses of systemic antifungal agents (itraconazole) and topical antifungal agents (2% miconazole shampoos and terbinafine-containing dusting powder). Diagnosis was confirmed by microscopic examination, culture, and punch biopsy. Due to the lack of response to standard therapy, posaconazole was initiated based on antifungal susceptibility testing (AFST). Marked clinical improvement was observed without adverse effects. This report documents a case of refractory dermatophytosis in which AFST informed the selection of posaconazole therapy. It highlights the diagnostic challenges of recurrent dermatophytosis, suggests that AFST-guided treatment strategies may help manage infections unresponsive to standard antifungal therapy, and demonstrates that posaconazole is a promising alternative antifungal agent for refractory dermatophytosis in dogs.

## Simple Summary

Dermatophytosis is a contagious superficial fungal skin disease in dogs and cats that can also be transmitted to humans. Veterinary dermatologists are increasingly encountering refractory cases that show poor response or recur despite appropriate antifungal therapy. This report presents an 8-year-old male Spitz dog with chronic dermatophytosis that did not respond to conventional systemic and topical antifungal treatments. Microscopic examination, fungal culture, antifungal susceptibility testing (AFST), and skin biopsy confirmed the diagnosis. Treatment with posaconazole resulted in marked clinical improvement and a complete clinical and mycological cure without adverse effects. This case demonstrates that posaconazole can be a safe and effective alternative for managing refractory dermatophytosis in dogs.

## 1. Introduction

Dermatophytosis is one of the most common superficial fungal infections in dogs and is usually responsive to systemic antifungal agents, such as itraconazole or terbinafine, combined with topical therapy [1]. In recent years, azole resistance and refractory or recurrent fungal cases have been described in veterinary medicine [2,3]. Contributing factors include inappropriate drug selection, inadequate treatment duration, poor owner compliance, and possible antifungal resistance [4]. Reports of multidrug-resistant dermatophyte isolates in companion animals remain limited but are growing, raising concerns about therapeutic challenges and potential zoonotic implications. In such refractory cases, antifungal susceptibility testing (AFST) is increasingly recommended to guide therapy, yet standardised treatment protocols for resistant infections in dogs are lacking. Posaconazole, a second-generation triazole antifungal, has a broad spectrum of activity and may serve as a salvage therapy in refractory cases [5]. The objective of this case report was to describe the successful management of refractory dermatophytosis in a Spitz dog using posaconazole after failure of conventional antifungal therapy.

## 2. Case Presentation

An eight-year-old intact male Spitz dog weighing 10 kg presented with an 11-month history of chronic dermatological lesions. The dog was primarily housed indoors with intermittent access to the garden and had no contact with other animals in the household. Notably, similar dermatological lesions were reported in two household members—a 20-year-old female and a 65-year-old female—suggesting possible zoonotic transmission.

**Figure 1:**
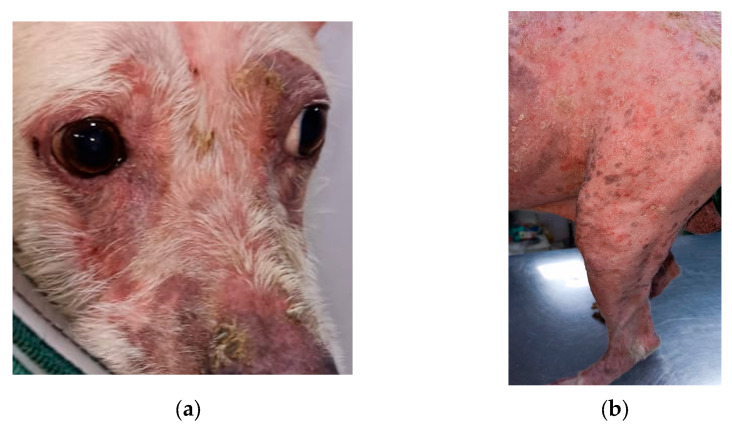
(**a**) Fascial dermatologic lesions at initial presentation, characterised by alopecia, erythema, and crusting. (**b**) Diffuse erythema with multifocal hyperpigmentation and alopecia on the dorsolateral trunk.

The dog was privately owned and had been referred for further diagnostic evaluation and management. Written informed consent for diagnostic procedures and treatment was obtained from the owner prior to inclusion in this report. Clinical examination revealed multifocal alopecia, erythema, scaling, and pruritus, predominantly involving the face, trunk, and limbs (Figure 1). The dog had initially been diagnosed with dermatophytosis and treated with oral itraconazole at 10 mg/kg body weight as a 1-month pulse therapy, along with topical antifungal therapy consisting of a 2% miconazole shampoo and terbinafine dusting powder. A relapse was observed four weeks after cessation of therapy, following which, the same treatment protocol was repeated multiple times by the pet owner without consultation. Subsequently, hepatotoxicity was detected, as evidenced by elevated alanine aminotransferase (ALT; 275 U/L) and alkaline phosphatase (ALP; 350 U/L) levels, whereas the complete blood count remained within normal limits, as mentioned in Table 1.

**Figure 2:**
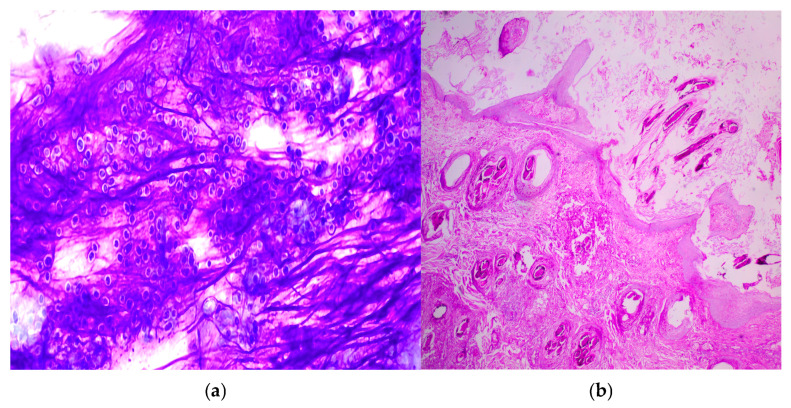
(**a**) Fungal spores observed on cytology (×100) using Diff-Quik stain. (**b**) Punch biopsy (PAS, ×10) showing fungal spores within the hair follicle.

**Figure 3:**
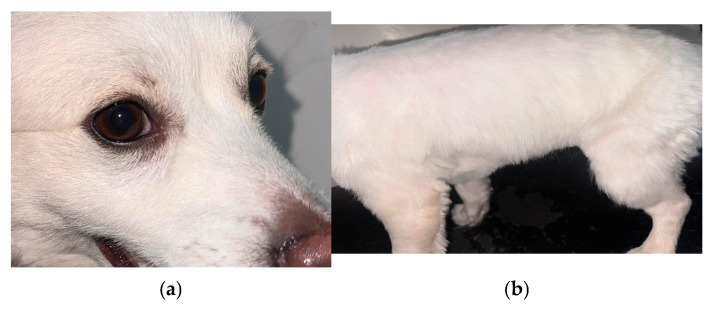
(**a**,**b**) Hair regrowth and resolution of cutaneous lesions after 8 weeks of posaconazole therapy.

Antifungal therapy was discontinued, and the dog was subsequently referred to our facility for further diagnostic evaluation and management. Upon presentation, hair samples were collected for fungal culture, and antifungal susceptibility testing was performed due to the patient’s history of therapeutic failure. Deep skin scrapings were negative for ectoparasites. A direct impression smear was prepared for cytological examination, which revealed multiple fungal spores. Skin biopsies were obtained to rule out underlying immune-mediated dermatological conditions. Histopathological examination demonstrated fungal spores within the hair follicles (Figure 2). Fungal culture yielded growth of *Microsporum canis* after 14 days of incubation. Antifungal susceptibility testing showed susceptibility to amphotericin B, posaconazole, and voriconazole, while resistance was noted to routinely used antifungal agents (Table 2). Prior to initiating further systemic antifungal therapy, serum biochemical parameters were reassessed, and ALT and ALP values were within the normal reference range. Based on the susceptibility results, posaconazole was administered orally at 10 mg/kg once daily for 3 weeks, followed by 5 mg/kg once daily for an additional 3 weeks (Poshope DR^®^, Abbott Healthcare Pvt. Ltd. Mumbai, India). Liver enzyme levels were monitored regularly throughout the treatment period. Adjunctive topical therapy with an essential fatty acid-based shampoo (Essential 6 Sebo Shampoo^®^, Dermoscent Laboratoire, Castres, France) was administered once weekly. Progressive clinical improvement was observed within five weeks of initiating posaconazole therapy, characterised by a marked reduction in erythema and scaling, followed by gradual hair regrowth (Figure 3). Hepatoprotective supplementation containing S-adenosyl methionine and silybin (Lisybin Medium^®^, SAVA Healthcare Limited, Pune, India) was administered during the course of posaconazole therapy and continued for two weeks after treatment discontinuation. At the completion of treatment, complete clinical resolution was achieved and repeat mycological examination yielded negative results.

## 3. Discussion

Canine dermatophytosis is a common, contagious, and zoonotic superficial fungal infection affecting keratinised tissues, such as hair, the *stratum corneum*, and claws. It is most frequently caused by *Microsporum canis*, *Microsporum gypseum*, and *Trichophyton mentagrophytes* complex. Although the majority of the cases respond well to appropriate systemic antifungal therapy combined with topical management and environmental decontamination, treatment failures, relapses, and chronic infections are being increasingly documented in clinical practice, particularly in cases with extensive disease, poor compliance, repeated empirical antifungal use, or underlying host and environmental factors [1]. There is currently no universally accepted definition of refractory dermatophytosis in veterinary medicine. Clinically, it can be defined as persistent or recurrent dermatophyte infection despite adequate duration and dosage of appropriate antifungal therapy, with confirmed owner compliance and environmental control, or failure to achieve mycological cure after repeated courses of standard antifungal agents [1,6]. Refractory disease warrants a more advanced diagnostic and therapeutic approach, including identification of fungal species, histopathology, and antifungal susceptibility testing (AFST). Diagnosis of dermatophytosis is typically based on clinical presentation, direct microscopy, wood’s lamp examination, fungal culture, and, in selected cases, histopathology. However, recurrent or refractory infections pose significant diagnostic challenges. Prior antifungal therapy can reduce the fungal load and lead to false-negative cultures, emphasising the importance of appropriate sampling from active lesion margins and, when indicated, skin biopsy with special stains such as periodic acid–Schiff (PAS) to demonstrate fungal elements within hair follicles and the *stratum corneum* [1,7]. Identification of dermatophyte species through fungal culture remains a critical step as different species may vary in pathogenicity, environmental persistence, zoonotic potential, and antifungal susceptibility profile [6]. In the present case, fungal culture and histopathology confirmed dermatophytosis after recurrent clinical disease, supporting the diagnosis of refractory dermatophytosis.

Antifungal susceptibility testing is not routinely recommended for uncomplicated dermatophytosis, as most infections respond to first-line agents such as itraconazole or terbinafine. Nevertheless, AFST becomes clinically relevant in refractory cases, especially when animals have been exposed to multiple antifungal drugs without sustained clinical or mycological cure [6]. In the present case, AFST demonstrated resistance to several commonly used antifungal agents, while the culture showed in vitro susceptibility to posaconazole. This finding justified selecting posaconazole as salvage therapy despite its limited routine use in veterinary dermatology. Posaconazole is a second-generation triazole antifungal agent with broad-spectrum activity against yeasts and fungi. It acts by inhibiting fungal cytochrome P450-dependent 14α-demethylase, thereby disrupting ergosterol synthesis and fungal cell membrane integrity [8]. The clinical response observed in this case is consistent with previous reports describing the broad-spectrum antifungal activity of posaconazole against dermatophytes. In vitro studies have demonstrated that posaconazole has a low minimum inhibitory concentration against *Microsporum* species, suggesting strong antifungal activity. Although veterinary clinical reports describing posaconazole use specifically for dermatophytosis are limited, successful outcomes have been reported in systemic mycoses such as histoplasmosis and cryptococcosis in dogs and cats that were refractory to conventional azole therapy [9,10]. These findings support the potential role of posaconazole as a salvage antifungal agent when standard treatments fail. In veterinary medicine, its use is limited, primarily due to cost, limited availability, and a lack of dermatology-specific clinical data. In vitro studies have demonstrated that posaconazole exhibits potent activity against dermatophyte isolates with minimum inhibitory concentrations comparable to or lower than those of itraconazole in some studies [11]. In human medicine, posaconazole is widely used for prophylaxis and treatment of invasive fungal infections and has been successfully used in severe, extensive, or treatment-resistant dermatophytosis, particularly in immunocompromised patients [12]. Together with AFST-guided susceptibility results, these data support the rational use of posaconazole in refractory canine dermatophytosis. Systemic azole antifungals are associated with potential hepatotoxicity, especially during prolonged therapy. Therefore, baseline and periodic monitoring of serum biochemistry, including liver enzymes, is recommended when posaconazole or other azoles are administered long term [1,11,13]. In the present case, routine haematology and serum biochemistry were performed to monitor hepatic function, and liver-supportive therapy was administered concurrently, which may have contributed to the favourable outcome.

Successful management of refractory dermatophytosis requires a multimodal approach that combines systemic antifungal therapy guided by susceptibility testing, topical therapy to reduce the fungal burden, and strict environmental hygiene. Topical therapy, including antifungal or essential fatty acid-based shampoos, plays an important role in decreasing surface contamination, reducing environmental shedding, and limiting zoonotic transmission [1,14]. Despite the favourable outcome observed in this case, several limitations should be acknowledged. First, the present report describes a single clinical case, which limits the generalisability of the findings to a broader population of dogs with dermatophytosis. Second, although antifungal susceptibility testing supported the use of posaconazole, standardised clinical breakpoints for dermatophytes in veterinary medicines remain limited. Additionally, the high cost and limited availability of posaconazole may restrict its routine clinical use. Further studies, including larger case series or controlled clinical trials, are required to better establish the efficacy, optimal dosing regimens, and safety profile of posaconazole in canine dermatophytosis.

Dermatophytosis is a recognised zoonosis. Infected animals may transmit the disease to humans, especially children, the elderly, and immunocompromised individuals. Therefore, owner education about hygiene, environmental cleaning, and avoidance of contact by those at high risk is essential and was emphasised in this case. 

In this case, posaconazole therapy, combined with topical management and liver support, led to marked clinical improvement within 4 weeks, with complete resolution by 8 weeks. Treatment continued beyond clinical resolution to reduce relapse risk. Although the cost of posaconazole remains a major limitation for long-term use, this case shows that AFST-guided posaconazole therapy can be effective and rational in refractory canine dermatophytosis. Moreover, this case highlights the importance of antifungal susceptibility testing in recurrent dermatophytosis and suggests that posaconazole may be valuable as salvage therapy when conventional antifungals fail.

This report highlights both strengths and limitations. A notable strength is the thorough diagnostic evaluation, incorporating fungal culture, histopathological examination, and antifungal susceptibility testing, which allowed for rational selection of therapy in a treatment-resistant case. The integration of systemic and topical interventions, along with consistent clinical follow-up, likely contributed to the successful clinical resolution and supports the value of an evidence-guided therapeutic approach. Nevertheless, the interpretation of these findings is limited by the single-case description, which does not permit extrapolation to the broader canine population. The scarcity of dermatology-focused data on posaconazole use in veterinary patients limits the clinical interpretation of susceptibility testing outcomes. Practical constraints, including the relatively high cost and restricted availability of posaconazole, may further limit its widespread application. Future investigations involving larger cohorts and controlled study designs are needed to more clearly establish the clinical efficacy, safety profile, and optimal treatment protocols for posaconazole in canine dermatophytosis.

## 4. Conclusions

This case shows that antifungal susceptibility testing is crucial for guiding treatment in refractory canine dermatophytosis. When selected based on susceptibility results and appropriate clinical monitoring, posaconazole could be an effective salvage option for dogs unresponsive to standard antifungals. Due to limited veterinary data, posaconazole should be reserved for refractory cases and guided by fungal culture and testing. Controlled clinical studies are needed to clarify its efficacy, safety, and optimal protocols in canine dermatophytosis.

## Figures and Tables

**Table 1:** Haematological and Biochemical Findings in the Dog with Refractory Dermatophytosis.

Parameter	Before Treatment	Reference Range
Haemoglobin (g/dL)	13.5	12–18
Total Leukocyte Count (×10^9^/L)	9.8	6–17
Alanine Aminotransferase (ALT) (U/L)	275	10–100
Alkaline Phosphatase (ALP) (U/L)	350	20–150
Total Protein (g/dL)	6.8	5.4–7.5
Albumin (g/dL)	3.4	2.6–4.0

**Table 2:** Antifungal susceptibility results and clinical treatment decisions.

Antifungal Drug	Susceptibility Result	Clinical Interpretation	Treatment Decision
Itraconazole	Resistant	Likely explains previous treatment failure	Not selected
Ketoconazole	Resistant	Reduced effectiveness expected	Not selected
Fluconazole	Resistant	Not suitable for therapy	Not selected
Amphotericin B	Susceptible	Potentially effective antifungal	Not chosen due to systemic toxicity concerns
Posaconazole	Susceptible	Effective antifungal with good activity against dermatophytes	Selected as systemic therapy
Voriconazole	Susceptible	Active antifungal agent	Not selected due to cost and limited veterinary dermatology data

## Data Availability

The original contributions presented in this study are included in the article. Further inquiries can be directed to the corresponding author.
